# Anti-PD-1 therapy achieves favorable outcomes in HBV-positive non-liver cancer

**DOI:** 10.1038/s41389-023-00468-0

**Published:** 2023-04-20

**Authors:** Jie Zhou, Guanming Chen, Jiuling Wang, Bo Zhou, Xuemin Sun, Jinsong Wang, Shu Tang, Xiangju Xing, Xiaofei Hu, Yang Zhao, Yu Peng, Wenjiong Shi, Tingting Zhao, Yuzhang Wu, Hanbing Zhong, Ni Hong, Zhihua Ruan, Yi Zhang, Wenfei Jin

**Affiliations:** 1 Chongqing International Institute for Immunology, 400030 Chongqing, China; 2 Department of Oncology, Southwest Hospital, Army Medical University, 400038 Chongqing, China; 3 School of Life Sciences, Southern University of Science and Technology, 518055 Shenzhen, China; 4 Institute of Immunology, PLA, Army Medical University, 400038 Chongqing, China; 5 Institute of Cancer, Xinqiao Hospital, Army Medical University, 400038 Chongqing, China; 6 Pulmonary and Critical Care Medicine, The Third Affiliated Hospital of Chongqing Medical University, 400038 Chongqing, China; 7 Department of Radiology, Southwest Hospital, Army Medical University, 400038 Chongqing, China; 8 School of Pharmacy and Bioengineering, Chongqing University of Technology, 400054 Chongqing, China

**Keywords:** Immunotherapy, Tumour immunology

## Abstract

Anti-PD-1 therapy has shown promising outcomes in the treatment of different types of cancer. It is of fundamental interest to analyze the efficacy of anti-PD-1 therapy in cancer patients infected with hepatitis B virus (HBV) since the comorbidity of HBV and cancer is widely documented. We designed a multicenter retrospective study to evaluate the efficacy of anti-PD-1 therapy on non-liver cancer patients infected with HBV. We found anti-PD-1 therapy achieved much better outcomes in HBV+ non-liver cancer patients than their HBV– counterparts. We performed single-cell RNA sequencing (scRNA-seq) on peripheral blood mononuclear cells (PBMCs) from esophageal squamous cell carcinoma (ESCC) patients. We found both cytotoxicity score of T cells and MHC score of B cells significantly increased after anti-PD-1 therapy in HBV+ ESCC patients. We also identified CX3CR1^high^ T_EFF_, a subset of CD8^+^ T_EFF_, associated with better clinical outcome in HBV+ ESCC patients. Lastly, we found CD8^+^ T_EFF_ from HBV+ ESCC patients showing higher fraction of Exhaustion^hi^ T than their HBV– counterpart. In summary, anti-PD-1 therapy on HBV+ non-liver cancer patients is safe and achieves better outcomes than that on HBV– non-liver cancer patients, potentially because HBV+ patients had higher fraction of Exhaustion^hi^ T, which made them more efficiently respond to anti-PD-1 therapy.

## Introduction

Immune checkpoint blockade (ICB) therapy is a promising immunotherapy approach, among which anti-programmed cell death-1 (anti-PD-1) therapy yielded promising outcomes in treatment of dozen of types of cancer, including melanoma, hepatocellular carcinoma (HCC), gastric cancer, and esophageal cancer [1–6]. However, only a fraction of cancer patients responded to anti-PD-1 therapy. Of note, the efficacy of anti-PD-1 therapy is affected by many factors including tumor microenvironment, commensal microbiota, antibiotics, steroids, and viral infections [3, 7–9]. In particular, about 54% of HCC cases were associated with hepatitis B virus (HBV) infection [10], and HCC patients with HBV infection were excluded in early anti-PD-1 therapy trials due to concerns about the reactivation of HBV and uncertain about immune microenvironment. However, enrollment of HCC patients with HBV infection showed that low and intermediate HBV-DNA level (<500 IU/mL) did not impact the efficacy and safety of anti-PD-1 therapy [1, 11], even HCC patients with high HBV-DNA level did not show increased incidence of HBV-associated hepatitis [12, 13].

Simultaneously, the success of anti-PD-1 in cancer therapy potentially indicates that anti-PD-1 might be effective for treating infectious diseases since chronic infection also showed high expression of PD1 and T-cell exhaustion [14]. Over 350 million people in the world and 70 million people in China were infected with HBV, which was one of the most common comorbidities with non-liver cancer [15]. Recently, many studies analyzed anti-PD-1 therapy on patients with comorbidity of non-liver cancer and HBV infection [16–26]. Some of these studies only focused on the safety of anti-PD-1 therapy due to concerns about HBV reactivation [16, 21, 25, 26]. Other studies analyzed the efficacy of anti-PD-1 therapy [17–20, 22–24], which only enrolled a few non-liver cancer patients with HBV virus load (<7 samples). Since these studies did not design HBV+non-liver cancer patients and their HBV– counterparts matched case-control study [17–19, 23], it is still unknown whether patients with comorbidity of non-liver cancer and HBV infection could achieve similar efficiency compared with their HBV– counterparts.

Here, we performed a multicenter retrospective study on 84 non-liver cancer patients, including 35 HBV+ patients and 49 HBV– patients. We further performed single-cell RNA sequencing (scRNA-seq) on six peripheral blood mononuclear cells (PBMCs) samples from three esophageal squamous cell carcinoma (ESCC) patients (pre- and post-anti-PD-1 therapy), to analyze their response to anti-PD-1 therapy. We further analyzed the differences in the immune microenvironment between HBV+ ESCC patients and HBV– ESCC patients to explore the mechanism underlying their different response to anti-PD-1 therapy.

## Results

### HBV– patients and HBV+ patients are well matched

**Fig. 1:**
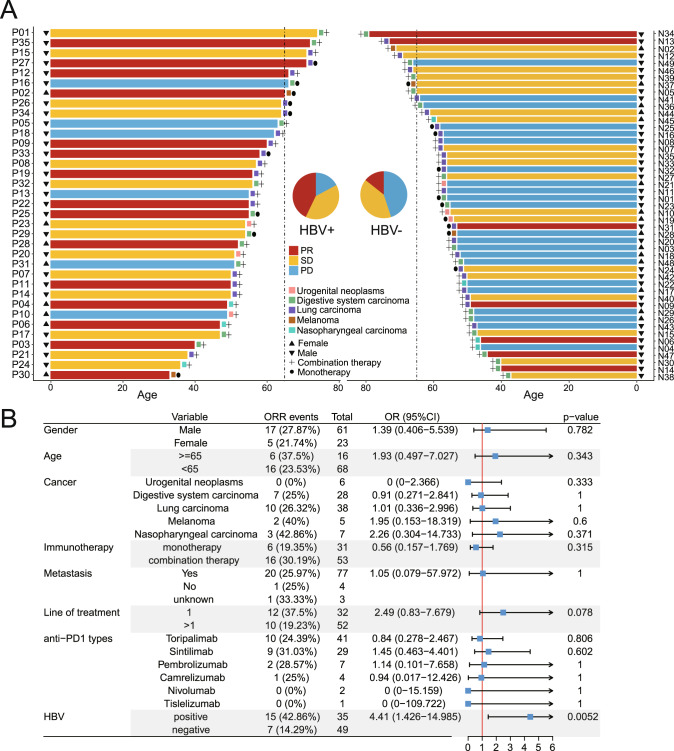
Clinical characteristics and efficacy of anti-PD-1 therapy in non-liver cancer patients with or without HBV infection. **A** HBV+ non-liver cancer patients and their HBV– counterparts were well matched for age, sex and cancer type. Each bar represents one patient’s information. Dashed line represents 65-year-old. **B** Forest plot of objective response rate (ORR) events. Odds ratio (OR) and *p*-value were calculated using Fisher’s exact test.

In order to analyze the effect of HBV infection on anti-PD-1 therapy in non-liver cancer patients, a total of 35 HBV+ non-liver cancer patients were screened from 7,231 cancer patients who visited during 2018–2021 (Fig. 1A, Supplementary Fig. S1A, Supplementary Table 1). We selected 49 HBV– non-liver cancer patients that matched HBV+ non-liver cancer patients in terms of cancer types, age, and gender (Fig. 1A, Supplementary Fig. S1A, Supplementary Table 2). The median age of HBV+ and HBV– non-liver cancer patients was 55-year-old (range: 33–74 years, with 7 patients ≥65) and 55-year-old (range: 37–79 years, with 9 patients ≥65), respectively. Both HBV+ and HBV– non-liver cancer patients were mainly males, with 27 males (77.1%) and 34 males (69.4%), respectively (Supplementary Table 3). In our study, 42.9% of HBV+ patients and 34.7% of HBV– patients received anti-PD-1 therapy as the first-line therapy, with no significant difference between the two groups (*p*-value = 0.499) (Supplementary Table 3).

### Anti-PD-1 therapy achieves favorable outcomes in HBV+ non-liver cancer patients

Surprisingly, anti-PD-1 therapy achieved significantly better outcomes in HBV+ non-liver cancer patients than in HBV– non-liver cancer patients (*p*-value = 0.0052), with 15 (42.86%) HBV+ non-liver cancer patients showing partial response (PR) while only 7 (14.29%) HBV– patients showing PR (Fig. 1B, Supplementary Tables 1–3). Moreover, 22 (44.9%) HBV– non-liver cancer patients developed progressive disease (PD), whereas only 6 (17.1%) HBV+ non-liver cancer patients developed PD.

Based on the objective response rate (ORR), the odds ratio (OR) of HBV+ non-liver cancer patients compared with their HBV– counterparts was 4.41 (95% CI; 1.426–14.985). HBV+ patients showed much longer overall survival (OS), further indicating that HBV+ patients have much better outcome than their HBV– counterparts (Fig. 1B, Supplementary Fig. S1B). Although these patients received different PD-1 antibody treatments, there was no significant difference in the outcome of different PD-1 antibodies (Fig. 1B). The outcomes of combination anti-PD-1 therapy showed higher ORR events (30.19%) than monotherapy (19.35%), although the difference is not significant (*p*-value = 0.315) (Fig. 1B) potentially due to the small sample size in this study. Furthermore, OR for first-line treatment compared with second- and beyond-line is 2.49 (95%CI; 0.83–7.679), which is not significant difference and was essentially consistent with a recent study by Hughes et al. [27].

### Change of population abundances in PBMCs after anti-PD-1 therapy

**Fig. 2:**
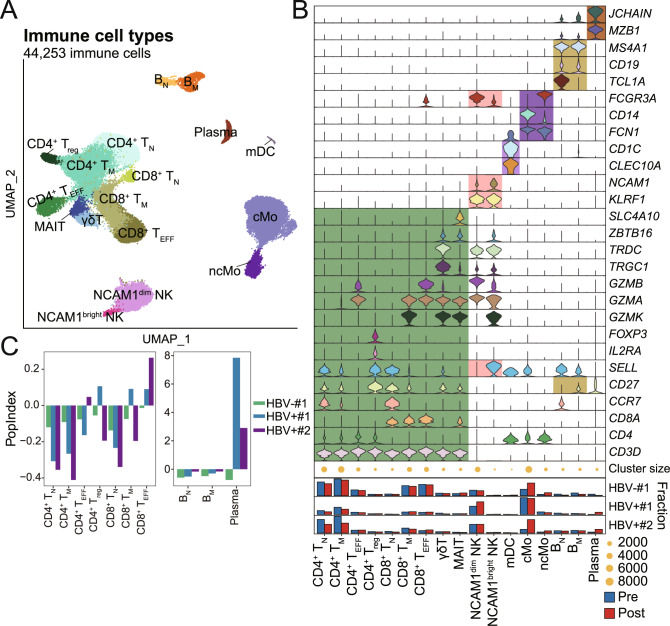
Cell subsets in PBMCs in 3 ESCC patients pre- and post-anti-PD-1 therapy. **A** UMAP projection of PBMCs from 3 ESCC patients with pre- and post-treatment samples, colored by cell type. Each dot represents a cell. **B** Marker genes, population size and fraction of cells pre-and post-anti-PD-1 therapy for each cell type in PBMC from the 3 ESCC patients. **C** Population change index (PopIndex) of T-cell subsets (left panel) and B-cell subsets (right panel) in PBMCs from the 3 ESCC patients pre- and post-anti-PD-1 therapy.

Three ESCC patients, with one HBV-negative patient (HBV–#1) and two HBV-positive patients (HBV+#1 and HBV+#2), were enrolled from the Southwest Hospital for exploring their response to anti-PD-1 therapy. HBV+#1 and HBV+#2, both with hepatitis B surface antigen (HBsAg)-positive and hepatitis B core antibody (HBcAb)-positive, had 1910 IU/mL HBV-DNA and 48,600 IU/mL HBV-DNA in blood, respectively (Supplementary Table 4). We performed 10x genomics scRNA-seq on the PBMCs of the three patients before anti-PD-1 therapy. All three patients received sintilimab (anti-PD-1, IBI 308) and paclitaxel liposome as first-line therapy. After one cycle of therapy (4 weeks), blood samples were collected from patients for routine blood test and scRNA-seq library preparation. HBV–#1 achieved stable disease (SD) to the anti-PD-1 therapy after one cycle of therapy. HBV+#1 and HBV+#2 achieved SD and PR, respectively. In total, 44,253 PBMCs from the 3 ESCC patients passed quality control and were clustered into 17 subsets that were annotated according to their specific expression of classic markers (Fig. 2A, B, Supplementary Fig. S2). The fractions of CD4^+^ T_N_, CD4^+^ T_M_, CD8^+^ T_N_, *B*_N_, and *B*_M_ consistently decreased across patients after anti-PD-1 therapy, while the fraction of cMo increased in both HBV–#1 and HBV+#2 (Fig. 2B). We developed population change index (PopIndex) to describe change of immune cell subsets pre- and post-anti-PD-1 therapy. We found the PopIndex of CD4^+^ T_EFF_, CD8^+^ T_EFF_ and plasma cells had pronounced increase in HBV+#2, which may contribute to partial response of HBV+#2 (Fig. 2C). These results were consistent with previous reports that increase of T_EFF_ or increase of plasma cells is correlated with tumor regression [28–30].

### Change of T-cell features and a T-cell subset after anti-PD-1 therapy

**Fig. 3:**
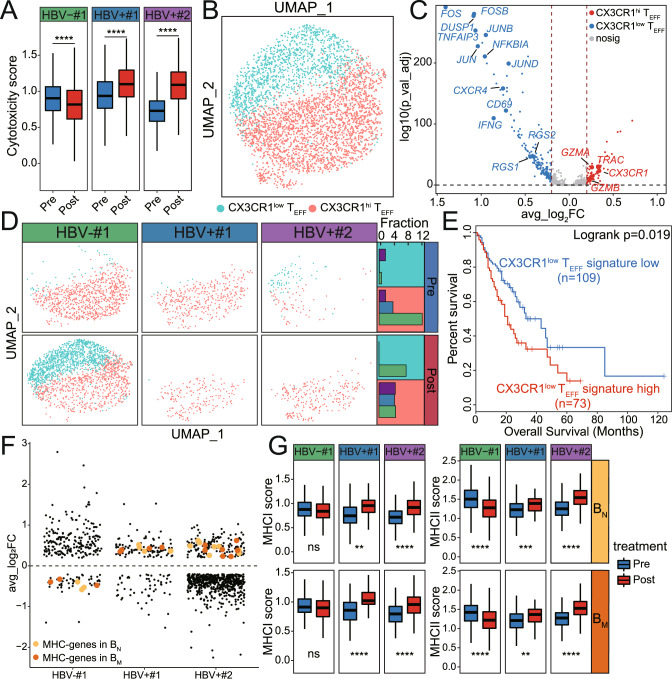
Change of immune cell features and subsets in the 3 ESCC patients. **A** Box plots of the cytotoxicity score of CD8^+^ T_EFF_ pre- and post-anti-PD-1 therapy in the three ESCC patients. Horizontal lines represent median values, with whiskers extending to the farthest data point within a maximum of 1.5× interquartile range. *p*-value was determined by Student’s *t*-test. ****p < 0.0001. **B** UMAP projection of CD8^+^ T_EFF_ cells, colored by cell subset. **C** Volcano plots of differentially expressed genes (DEGs) between CX3CR1^low^ and CX3CR1^hi^. The CX3CR1^low^-specific genes and CX3CR1^hi^-specific genes (Bonferroni-adjusted *p*-values < 0.05 and average Log_2_(fold-change) (avg_log_2_FC) > 0.2) were colored by blue and red, respectively. **D** UMAP projection of CD8^+^ T_EFF_ cells in each sample, colored by cell subset (left panel), and fraction of each subset in the three ESCC patients (right panel). **E** Survival curves of CX3CR1^low^ T signature high patients and other patients in ESCA samples from TCGA (*n* = 182). ESCA patients were divided into CX3CR1^low^ T signature high patients and other patients on 60% cut-off. *p*-value was determined by two-tailed log-rank test. **F** Jitter dot plots of DEGs pre- and post-anti-PD-1 therapy in the two B-cell subsets in each patient (Bonferroni-adjusted *p*-value < 0.05 and avg_log_2_FC > 0.2). Orange dots and red dots represent MHC-associated genes in *B*_N_ and *B*_M_, respectively. **G** Box plots of the MHC I score and MHC II score of *B*_N_ and *B*_M_ in three ESCC patients. *p*-value was calculated by Student’s *t*-test. ****p < 0.0001; **p < 0.01; ns, no significantly.

To analyze the effect of anti-PD-1 therapy on cell state of T cells, we identified the significantly differentially expressed genes (DEGs) between samples pre- and post-anti-PD-1 therapy in each of the 6T cell subsets, namely CD4^+^ T_N_, CD4^+^ T_M_, CD4^+^ T_EFF_, CD8^+^ T_N_, CD8^+^ T_M_, and CD8^+^ T_EFF_ (Supplementary Fig. S3A, Supplementary Table 5). GO enrichment analyses showed that cytokine signaling and T-cell activation signaling was significantly enriched in HBV+ ESCC patients, particularly in CD8^+^ T_EFF_ (Supplementary Fig. S3B, Supplementary Table 5) [31]. We further found that cytotoxicity scores of CD8^+^ T_EFF_ increased significantly after anti-PD-1 therapy in HBV+ ESCC patients, with HBV+#2 showing the most pronounced increase, while cytotoxicity scores of CD8^+^ T_EFF_ significantly decreased in HBV–#1 (Fig. 3A).

We further analyzed CD8^+^ T_EFF_ since it has the highest increase of population size and the highest increase of cytotoxicity score in HBV+#2. We clustered CD8^+^ T_EFF_ and identified two subsets, namely CX3 CR1^hi^ T_EFF_ and CX3 CR1^low^ T_EFF_ (Fig. 3B). Particularly, CX3CR1, a marker of T-cell differentiation, was identified as a predictive biomarker in response to ICB therapy [32, 33]. CX3CR1^hi^ T_EFF_ highly expressed cytotoxicity-associated genes such as *GZMA* and *GZMB* (Fig. 3C, Supplementary Table 6), consistent with recent reports that CX3CR1^+^ T_EFF_ lowly expressed co-inhibitor and highly expressed cytotoxicity-associated genes [32, 33]. In contrast, CX3CR1^low^ T_EFF_ highly expressed activator protein-1 (AP-1) family genes (*FOS*, *FOSB*, *JUNB*, *JUN* and *JUND*), which were cancer-related transcription factors [34]. CX3CR1^low^ T_EFF_ highly expressed regulators of G protein signaling (*RGS1* and *RGS2*) that was related to lower infiltration to cancer [35]. It also highly expressed *CXCR4* (Fig. 3C) that was correlated with T cells migration and promoted tumorigenesis [36]. These results revealed opposite functions of CX3CR1^low^ T_EFF_ and CX3CR1^hi^ T_EFF_. CX3CR1^low^ T_EFF_ was mainly enriched in HBV–#1 after anit-PD1 therapy, while CX3CR1^hi^ T_EFF_ were mainly in HBV+patient after anit-PD1 therapy (Fig. 3D), thus the enrichment of CX3CR1^hi^ T_EFF_ is associated with response to anti-PD-1 therapy. We performed survival analysis in esophagus carcinoma (ESCA) patients in The Cancer Genome Atlas (TCGA) [37]. We found the ESCA patients highly expressed CX3CR1^low^ T_EFF_-specific genes, namely CX3CR1^low^ T_EFF_ signature high patients, had lower survival than CX3CR1^low^ T_EFF_ signature low patients (Fig. 3E). Furthermore, CX3CR1^low^ T_EFF_ signature high patients displayed lower survival than CX3CR1^low^ T_EFF_ signature low patients in BLCA, LUSC, OV, STAD and COAD, supporting that CX3CR1^low^ and CX3CR1^hi^ T_EFF_ have different function in patients (Supplementary Fig. S4).

### Change of B/plasma cell features and subsets after anti-PD-1 therapy

Recent studies demonstrated that B cells played an important role in antitumor responses, which presented tumor-derived antigens to T cells and provoke T cells to regress tumor [28]. Therefore, we identified DEGs of each B-cell subset pre- and post-anti-PD-1 therapy in each patient. We found that major histocompatibility complex (MHC)-associated genes were significantly increased in *B*_N_ and *B*_M_ in HBV+ patients after anti-PD-1 therapy, while it significantly decreased in HBV–#1 (Fig. 3F and Supplementary Table 7). Furthermore, we found MHC I score and MHC II score of *B*_N_ and *B*_M_ in HBV+ patients significantly increased after anti-PD-1 therapy, while there is no change or decrease in HBV–#1 (Fig. 3G). In addition, many immunoglobulin genes such as *IGHG3, IGHG4, IGHG1, IGHM, IGLC2, IGLC1* and *IGKC* were up-regulated in HBV+ ESCC patients and down-regulated in HBV–#1 after anti-PD-1 therapy (Supplementary Fig. S5, Supplementary Table 8). Therefore, B cells showed a higher MHC score and higher expression of immunoglobulin genes in the 2 HBV+ ESCC patients after anti-PD-1 therapy.

### HBV+ non-liver cancer patients have higher fraction of Exhaustion^hi^ T before treatment

**Fig. 4:**
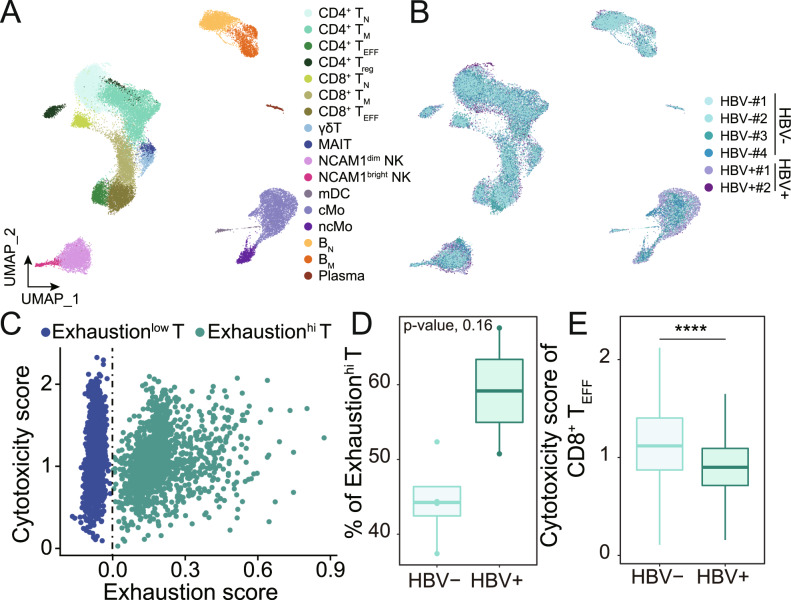
T cells from HBV+ non-liver cancer patients have been subjected to stronger immunosuppression than their HBV– counterparts. **A** UMAP projection of PBMCs from 6 ESCC patients, colored by cell type. Each dot represents a cell. **B** UMAP projection of PBMCs from 6 ESCC patients, colored by patient. Each dot represents a cell. **C** Scatter plot of the cytotoxicity score and exhaustion score of CD8^+^ T_EFF_ showed two distinct subsets, namely Exhaustion^hi^ T and Exhaustion^low^ T. Vertical dashed line represents exhaustion score = 0. **D** Box plot of the fraction of Exhaustion^hi^ T in CD8^+^ T_EFF_ in HBV– non-liver cancer patients and HBV+ non-liver cancer patients. *p*-value was determined by Kruskal–Wallis test. **E** Box plots of cytotoxicity score of CD8^+^ T_EFF_ in HBV– non-liver cancer patients and HBV+ non-liver cancer patients. *p*-value was determined by Student’s *t*-test.

Our analyses showed that immune function of HBV+ ESCC patients was significantly enhanced after anti-PD-1 therapy, while HBV– patient had little change, which might be caused by prevailing differences in the immune microenvironment between HBV+ patients and HBV– patients. We conducted a systemic comparison of the immune microenvironment between HBV+ ESCC patients and HBV– ESCC patients by integrating scRNA-seq data from Dinh et al. [38]. A total of 40,434 cells from 6 untreated ESCC patients were projected into UMAP plot (Fig. 4A, B). Interestingly, the distribution of exhaustion scores of CD8^+^ T_EFF_ showed there are two CD8^+^ T_EFF_ subsets (Fig. 4C), namely Exhaustion^hi^ T and Exhaustion^low^ T based on whether the normalized exhaustion score is >0 or not. HBV+ ESCC patients had much higher fraction of Exhaustion^hi^ T than HBV– ESCC patients (Fig. 4D). In addition, the cytotoxicity scores in CD8^+^ T_EFF_ in HBV+ ESCC patients were significantly higher than that in HBV– ESCC patients (Fig. 4E). We further analyzed 16 HBV infected patients from Zhang et al. [39], and found CD8T_c03-CX3CR1 subset reported in their study has the highest similarity to CD8^+^ T_EFF_ in this study (Supplementary Fig. S6A). We found the proportion of Exhaustion^hi^ T in CD8T_c03-CX3CR1 in HBV+ patients was much higher than HBV– individuals (Supplementary Fig. S6B–D). These results are not affected by anti-PD-1 therapy since we only analyzed the samples before therapy. Since anti-PD-1 therapy is mainly targeted at exhausted T cells and other immunosuppressed immune cells, thus a higher fraction of Exhaustion^hi^ T might provide more therapeutic targets, which could explain anti-PD-1 therapy is more effective in HBV+ non-liver cancer patients.

## Discussion

Different from these studies focusing on HBV infection and HCC comorbidity [1, 11, 13, 18, 40], this study focuses on HBV infection and non-liver cancer comorbidity. Our analyses showed patients with HBV+ non-liver cancer did not show increased irAEs than their HBV– counterparts during anti-PD-1 therapy. Intriguingly, HBV+ non-liver patients responded to anti-PD-1 therapy much better than their HBV– counterparts. Similar phenomena have been reported in other virus infections and cancer comorbidities. For example, either patients with Epstein–Barr virus infection and gastric cancer comorbidity, or patients with Epstein–Barr virus infection and non-Hodgkin lymphoma comorbidity, responded better to anti-PD-1 therapy than their Epstein–Barr virus negative counterparts [3, 41]. Finally, our results suggested that anti-PD-1 therapy should be encouraged to treat HBV+ non-liver cancer patients since they could achieve favorable outcome.

To explore the potential mechanism underlying the enhanced response of HBV+ non-liver cancer patients to anti-PD-1 therapy, we performed scRNA-seq on PBMCs and compared the features of each cell subset pre- and post-therapy in the 3 ESCC patients. We found cytotoxicity scores and MHC scores were significantly increased in CD8^+^ T_EFF_ and B cells in HBV+ ESCC patients after anti-PD-1 therapy, respectively. The enhanced immune function of PBMCs in HBV+ ESCC patients after anti-PD-1 therapy suggested that there is a stronger immunosuppressive microenvironment in HBV+ non-liver cancer patients before anti-PD-1 therapy. We indeed found that HBV+ ESCC patients had a higher fraction of Exhaustion^hi^ T compared with HBV– patients by integrating public data. We further found the HBV+patients had a higher fraction of Exhaustion^hi^ T than HBV– individuals with another 16 samples. Our observation that HBV+ non-liver cancer patients are subject to stronger immunosuppression are reasonable since it is well-known that both HBV infection [14, 42] and cancer [43] can lead immunosuppression and T-cell exhaustion. A higher fraction of Exhaustion^hi^ T in HBV+ non-liver cancer patients may lead to an enhanced response to anti-PD-1 therapy.

Despite the limited sample size of this study, our clinical data provided solid evidence to support the notion that anti-PD-1 therapy is a fairly good treatment for non-liver cancers. Our scRNA-seq data generated from the 3 ESCC patients provided important clues about the changes of immune cells. We compared the exhaustion scores of CD8^+^ T_EFF_ between HBV+ ESCC patients and HBV– ESCC patients and provided biological insight into their differential. In conclusion, we found anti-PD-1 therapy achieved better outcomes in non-liver cancer patients with HBV infection. We also found HBV+ non-liver patients had a higher fraction of Exhaustion^hi^ T, which could explain the differential response to anti-PD-1 between HBV+ and HBV– non-liver cancer patients.

## Methods

### Study design and patients

We designed a multicenter retrospective study to evaluate the efficacy of anti-PD-1 therapy on HBV+ non-liver cancer patients by comparing them with their HBV– counterparts (Supplementary Fig. S1A). This study was approved by IRB at the Southwest Hospital, AMU (ID: KY2021112). A total of 84 eligible non-liver cancer patients (35 HBV+ patients and 49 matched HBV– patients) were screened from 7231 cancer patients in clinical database of the Southwest Hospital, Xinqiao Hospital and the Third Affiliated Hospital, CQMU from 2018 to 2021 (Supplementary Fig. S1A).

The 35 HBV+ patients are HBsAg-positive (35; 100%) and HBcAb-positive (33; 94.3%). Furthermore, HBV-DNA was detected in all the 35 HBV+ patients, indicating HBV was active in those patients (Supplementary Table 1). Clinical data was collected for each patient, including age, gender, cancer types, metastasis, medical history, and therapy history. HBV+ non-liver cancer patients and HBV– non-liver cancer patients had matched for tumor types, including lung carcinoma, digestive system carcinoma, nasopharyngeal carcinoma, urogenital neoplasms, melanoma, and esophageal carcinoma. The 49 HBV– non-liver cancer patients were selected to match the 35 HBV+ non-liver cancer patients in regard to cancer types, age, and gender, thus the features of HBV– patients were similar to that of HBV+ patients (Supplementary Fig. S1A, Supplementary Tables 1–3).

### Anti-PD-1 therapy

Overall, 26 out of 35 HBV+ non-liver cancer patients received anti-PD-1 combination therapy (anti-PD-1 plus chemotherapy), while the other 9 patients received anti-PD-1 monotherapy. The anti-PD-1 antibodies used in this study include Toripalimab, Sintilimab, Pembrolizumab, Camrelizumab, Nivolumab, and Tislelizumab. Similarly, 39 out of the 49 HBV– non-liver cancer patients received anti-PD-1 combination therapy, while the other 10 patients received anti-PD-1 monotherapy. In addition, some patients were followed up for obtaining survival information after treatment (Supplementary Tables 1–3).

Patients enrolled in this study did not show any immune-related adverse event (irAE) or low grade (1 grade or 2 grade) irAEs during and after anti-PD-1 therapy, except three 3-grade irAEs, namely maculopapular rash in a HBV+ patient, which may be caused by drug treatment [44], cardiotoxicity in a HBV– patient and interstitial pneumonia in a HBV– patient. None of the patients died of irAEs or developed HBV-associated disease (Supplementary Tables 1–3). Overall, irAEs in HBV+ non-liver cancer patients were not significantly different from that in their HBV– counterparts, consistent with recent reports that anti-PD-1 therapy was safe for treatment of HBV+ non-liver cancer patients [17, 18, 40].

### scRNA-seq library preparation and sequencing

We performed scRNA-seq of 6 PBMCs samples derived from the three ESCC patients. This study was approved by IRB at the Southwest Hospital, AMU (ID: KY2021112). All individuals signed an informed consent form approved by the IRBs at the time of enrollment. Patients received the treatment according to clinical routine. The scRNA-seq library preparation and data analyses following our previous studies [45–47].

Approximately 5 mL blood sample was phlebotomized from each of the three ESCC patients pre- and post-treatment and was transported to the laboratory in an EDTA- containing anticoagulant tube (WEGO, Blood Collection Tube with Vacuum for Single Use, EDTA-K2). Blood samples were centrifuged at 1750 rpm for 8 min to collect the white blood cells. Lymphocyte Separation Medium (Human) (TBD, LTS1077) was added to substratum cell precipitation to obtain PBMCs after centrifugating at 1950rpm for 20 min and washed in 0.04% BSA of PBS (Gibco PBS pH7.4 basic 1x, 8121456) for twice with centrifuging at 1000 rpm for 5 min. Trypan blue (Beyotime Trypan blue 10 mL, C360I-2) staining was used to check for cell viability. The single-cell RNA sequencing libraries of PBMCs were generated using Chromium Next GEM Single-Cell 3’ Reagent Kit v3.1 from 10x genomics. Briefly, 20,000 cells were loaded into each channel of the 10x Chromium controller to generate single-cell GEMs. Single-cell RNA-seq libraries were prepared using the Chromium Single-Cell 3’ Gel Bead and Library Kit (10x Genomics), according to manufacturers’ instructions. Sequencing libraries were loaded at 2.4 pM on an Illumina NovaSeq 6000 with 2× 75 paired-end kits.

### Single-cell RNA-seq data pre-process

Briefly, we mapped the reads to the human reference genome GRCh38 and aggregated the gene expression matrixes using Cell Ranger (V.5.0.0). We constituted a Seurat object using the filter expression matrixes from Cell Ranger. Seurat package (V.3.2.3) [48] and Harmony package (V.1.0) [49] were used for the following analyses. Genes expressed in <20 cells were filtered out. Low-quality cells were filtered out if they met the criteria: (1) <200 features number; (2) <800 UMI number; or (3) >15% mitochondrial genome UMIs. We further normalized the gene expression matrix using the *NormalizeData* function. We identified the top 3,000 highly variable genes using the *FindVariableFeatures* function and further scaled the gene expression matrix using the *ScaleData* function. We conducted principal component analysis (PCA) for linear dimension reduction using the *RunPCA* function and the top 30 PCs were used for further analyses. The *RunHarmony* function in Harmony package was used to remove the batch effect in our data. Finally, Uniform Manifold Approximation and Projection for Dimension Reduction (UMAP) were conducted using the *RunUMAP* function.

### Clustering and identification of cell subsets

The cell clusters were inferred using *FindNeighbors* and *FindClusters* functions based on the normalized gene expression matrix. We identified 17 clusters that were annotated according to their specific expression of classic markers, including 9T cell subsets (*CD3D*), 2 natural killer cell (NK) subsets (*KLRF1*), 2 monocyte subsets (*FCN1*), 2 B-cell subsets (*CD19*), mDC (*CLEC10A*) and plasma cell (*MZB1*) (Supplementary Fig. S2B). The 2 NK cell subsets are *NCAM1*^bright^ NK and *NCAM1*^dim^ NK. The 2 B-cell subsets are naive B cells (*B*_N_) (*TCL1A*^*+*^) and memory B cells (B_M_) (*CD27*^*+*^). The 2 monocyte subsets are classic monocyte (cMo) (*CD14*^+^) and non-classical Monocyte (ncMo) (*FCGR3A*^*+*^). The 9T cell subsets are CD4^+^ Naive T cells (CD4^+^ T_N_) (*CD4*^*+*^ *CCR7*^*+*^), CD8^+^ Naive T cells (CD8^+^ T_N_) (*CD8*^*+*^ *CCR7*^*+*^), CD4^+^ memory T cells (CD4^+^ T_M_) (*CD4*^*+*^ *SELL*^*lo*^ *CCR7*^*lo*^), CD8^+^ memory T cells (CD8^+^ T_M_) (*CD8*^*+*^ *SELL*^*lo*^ *CCR7*^*lo*^), CD4^+^ effector T cells (CD4^+^ T_EFF_) (*CD4*^*+*^ *GZMA*^*+*^ *GZMB*^*+*^), CD8^+^ effector T cells (CD8^+^ T_EFF_) (*CD8*^*+*^ *GZMA*^*+*^ *GZMB*^*+*^), CD4^+^ regulatory T cells (CD4^+^ T_reg_) (*CD4*^*+*^ *FOXP3*^*+*^), γδT (*TRDC*^*+*^ *TRGC1*^*+*^) and mucosal-associated invariant T-cell (MAIT) (*ZBTB16*^*+*^ *SLC4A10*^*+*^) (Fig. 2A, B).

### Comparison of population size and DEGs

We calculated the PopIndex of each cell subset to measure the relative change of population size after anti-PD-1 therapy. The PopIndex was defined as PopIndex = *(F*_post_ *–* *F*_pre_*)/F*_pre_, where *F*_pre_ is the fraction of a cell subset in PBMCs before anti-PD-1 therapy, and *F*_post_ is the fraction of the same cell subset in PBMCs after anti-PD-1 therapy. A positive value of PopIndex indicates the increase of population size after anti-PD-1 therapy, and a negative value indicates population size decrease after anti-PD-1 therapy.

The *FindMarkers* function was utilized to find DEGs between the cell subsets. DEGs with bonferroni-adjusted *p*-values < 0.05 and average Log_2_ (fold-change) (avg_log_2_FC) > 0.2 were kept for further analyses.

### Comparison of functional features for each cell subset pre- and post-anti-PD-1 therapy

In order to evaluate the changes of functional features of each cell subset pre- and post-anti-PD-1 therapy, we calculated the cytotoxicity score, exhaustion score, major histocompatibility complex (MHC)-I score and MHC-II score of each cell using the *AddModuleScore* function. All the scores were measured according to expression of specific functional genes. For instance, the cytotoxicity score was calculated based on 12 cytotoxicity-associated genes: *PRF1, IFNG, GNLY, NKG7, GZMB, GZMA, GZMH, KLRK1, KLRB1, KLRD1, CTSW*, and *CST7*. The exhaustion score was calculated based on 6 exhaustion-associated genes: *LAG3, TIGIT, PDCD1, CTLA4, HAVCR2* and *TOX*. The MHC-I score was calculated based on 9 MHC-I-associated genes: *HLA-A, HLA-B, HLA-C PSMB8, PSMB9, TAP1, TAP2, TAPBP* and *B2M*. The MHC-II score was calculated based on 11 MHC-II-associated genes: *HLA-DRA, HLA-DRB1, HLA-DRB5, HLA-DPA1, HLA-DPB1, HLA-DQA1, HLA-DQA2, HLA-DQB1, HLA-DQB2, HLA-DMA*, and *HLA-DMB*.

### Statistical analyses

The OR value and *p*-value of clinical statistical analyses were calculated using Fisher’s exact test. The statistical significance of the score of gene sets was determined by Student’s *t*-test. Log-rank-test was utilized to calculate the *p*-value of the survival analyses. Kruskal–Wallis test was used to determine whether the proportion of Exhaustion^hi^ T were significantly different between two samples.

## Supplementary information

Supplementary figure

Table S1

Table S2

Table S3

Table S4

Table S5

Table S6

Table S7

Table S8

## Data Availability

The sequencing data have been deposited in Genome Sequence Archive in BIG Data Center under the accession number HRA002492.

## Code availability

Code used for analyses is available upon request.
