# Supplementary material for: Anti-PD-1 therapy achieves favorable outcomes in HBV-positive non-liver cancer
Source: Oncogenesis. 2023 Apr 20;12(1):22. doi: 10.1038/s41389-023-00468-0 (PMC10119302; doi:10.1038/s41389-023-00468-0)
Supplement: Supplementary file 1 — Supplementary figure [file 41389_2023_468_MOESM1_ESM.pdf]

Supplementary Figures

Anti-PD-1 therapy achieves favourable outcomes in HBV-positive non-liver cancer

Jie Zhou[1, 2#], Guanming Chen[3#], Jiuling Wang[1, 4#], Bo Zhou[3], Xuemin Sun[1, 4], Jinsong Wang[1, 4], Shu Tang[5], Xiangju Xing[6], Xiaofei Hu[7], Yang Zhao[2], Yu Peng[2], Wenjiong Shi[1], Tingting Zhao[1, 8], Yuzhang Wu[4], Hanbing Zhong[3], Ni Hong[3], Zhihua Ruan[2*], Yi Zhang[1, 8*], Wenfei Jin[3*]

1. Chongqing International Institute for Immunology, Chongqing, 400030, China

2. Department of Oncology, Southwest Hospital, Army Medical University, Chongqing, 400038, China

3. School of Life Sciences, Southern University of Science and Technology, Shenzhen, 518055, China

4. Institute of Immunology, PLA, Army Medical University, Chongqing, 400038, China

5. Institute of Cancer, Xinqiao Hospital, Army Medical University, Chongqing, 400038, China

6. Pulmonary and Critical Care Medicine, The Third Affiliated Hospital of Chongqing Medical University, Chongqing, 400038, China

7. Department of Radiology, Southwest Hospital, Army Medical University, Chongqing, 400038, China

8. School of Pharmacy and Bioengineering, Chongqing University of Technology, Chongqing, 400054, China

# Equal contribution

*Corresponding author: Zhihua Ruan, E-mail: rzh1234@163.com or Yi Zhang, E-mail: zy509419@foxmail.com or Wenfei Jin, E-mail: jinwf@sustech.edu.cn

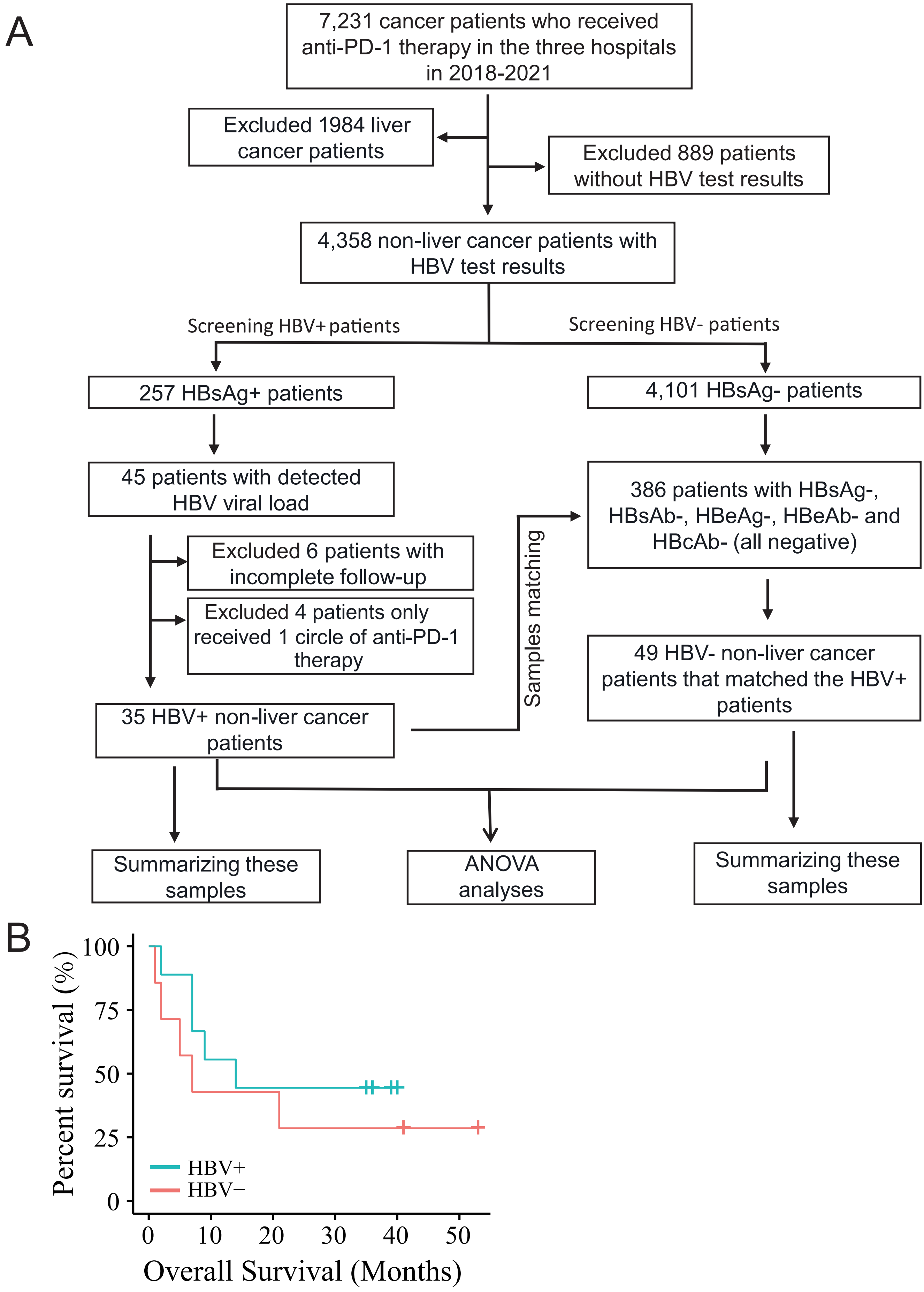

Fig S1. Sample screening and survival curves of patients.

A. Study flowchart detailing sample screening in this retrospective study. The flow of enrolled patients.

B. Survival curves of HBV+ (n=9) and HBV- (n=7) non-liver cancer patients.

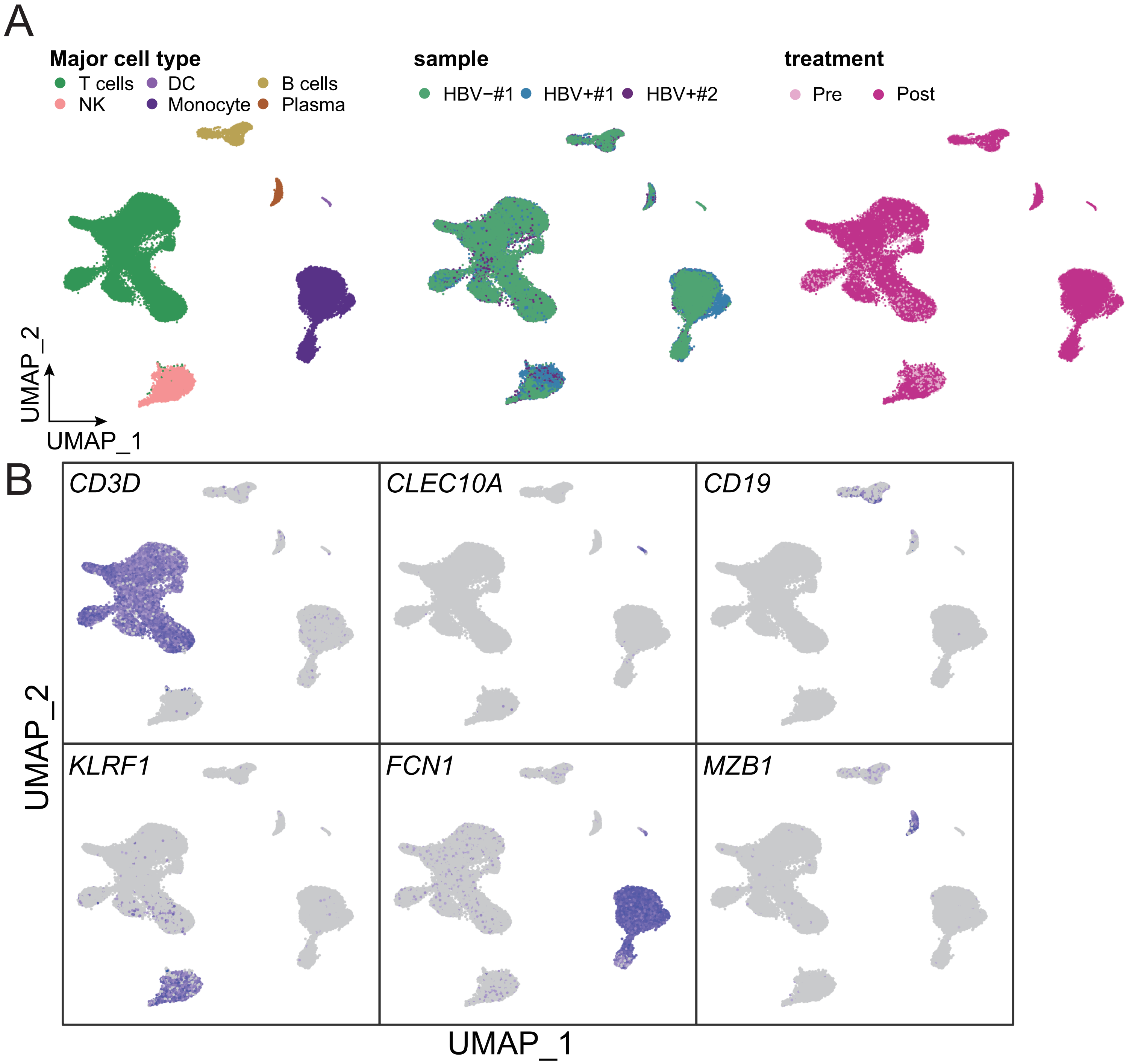

Fig S2. Atlas of PBMCs based on the 3 ESCC patients.

A. UMAP projection of PBMCs from the 3 ESCC patients pre- and post-anti-PD-1 therapy, colored by major cell types (left panel); colored by patients (middle panel); or colored by pre- or post-anti-PD-1 therapy (right panel).

B. UMAP projection of PBMCs, with each cell colored based on their normalized expression of *CD3D, CLEC10A, CD19, KLRF1, FCN1* and *MZB1*, respectively.

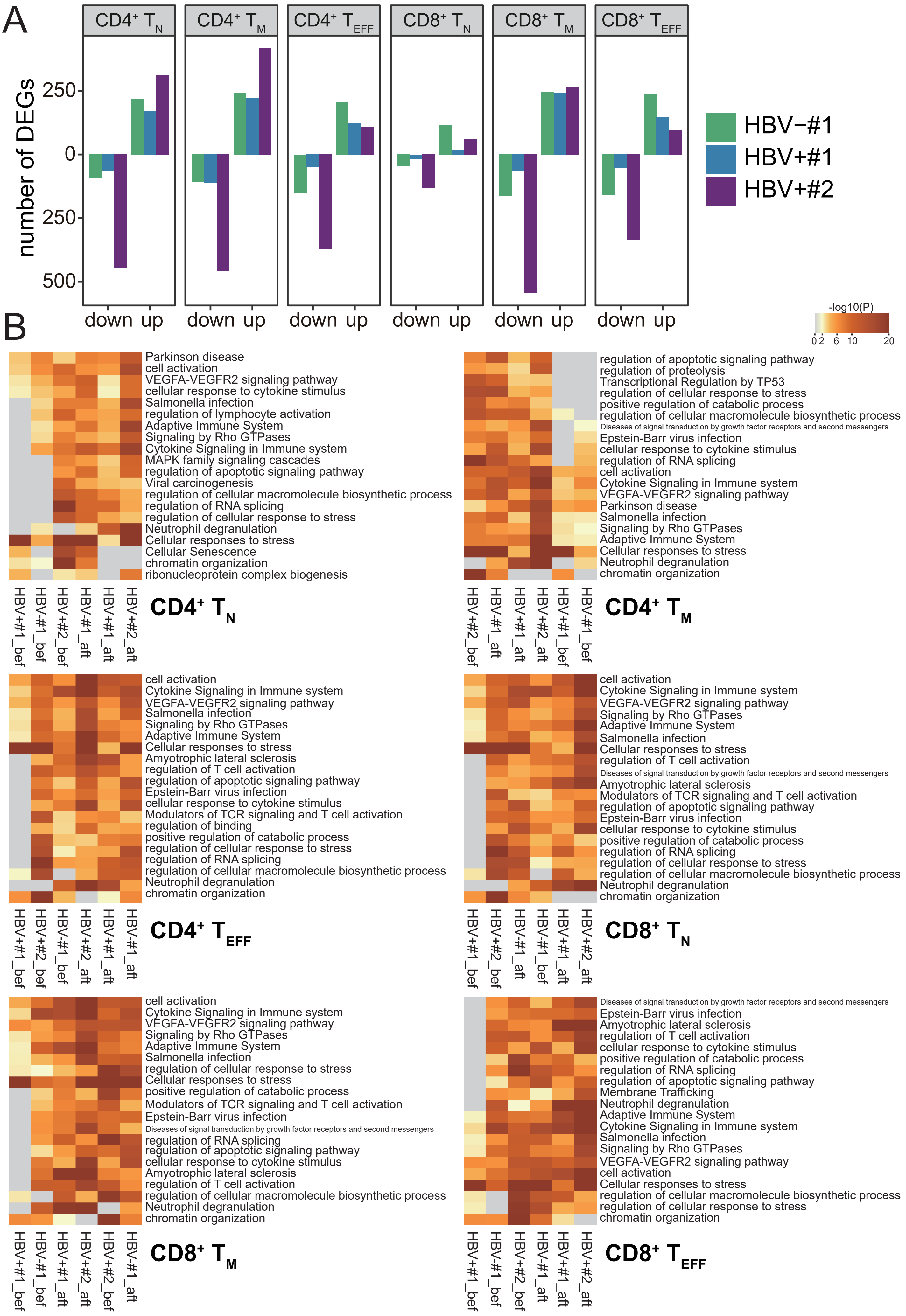
A
CD4+ TN
CD4+ TM
CD4+ TEFF
CD8+ TN
CD8+ TM
CD8+ TEFF
number of DEGs
250
0
250
500
down up
HBV−#1
HBV+#1
HBV+#2
B
-log10(P)
0 2 6 10 20
Parkinson disease
cell activation
VEGFA-VEGFR2 signaling pathway
cellular response to cytokine stimulus
Salmonella infection
regulation of lymphocyte activation
Adaptive Immune System
Signaling by Rho GTPases
Cytokine Signaling in Immune system
MAPK family signaling cascades
regulation of apoptotic signaling pathway
Viral carcinogenesis
regulation of cellular macromolecule biosynthetic process
regulation of RNA splicing
regulation of cellular response to stress
Neutrophil degranulation
Cellular responses to stress
Cellular Senescence
chromatin organization
ribonucleoprotein complex biogenesis
HBV+#1_bef HBV-#1_bef HBV+#2_bef HBV-#1_aft HBV+#1_aft HBV+#2_aft
CD4+ TN
regulation of apoptotic signaling pathway
regulation of proteolysis
Transcriptional Regulation by TP53
regulation of cellular response to stress
positive regulation of catabolic process
regulation of cellular macromolecule biosynthetic process
Diseases of signal transduction by growth factor receptors and second messengers
Epstein-Barr virus infection
cellular response to cytokine stimulus
regulation of RNA splicing
cell activation
Cytokine Signaling in Immune system
VEGFA-VEGFR2 signaling pathway
Parkinson disease
Salmonella infection
Signaling by Rho GTPases
Adaptive Immune System
Cellular responses to stress
Neutrophil degranulation
chromatin organization
HBV+#2_bef HBV-#1_aft HBV+#1_aft HBV+#2_aft HBV+#1_bef HBV-#1_bef
CD4+ TM
cell activation
Cytokine Signaling in Immune system
VEGFA-VEGFR2 signaling pathway
Salmonella infection
Signaling by Rho GTPases
Adaptive Immune System
Cellular responses to stress
Amyotrophic lateral sclerosis
regulation of T cell activation
regulation of apoptotic signaling pathway
Epstein-Barr virus infection
cellular response to cytokine stimulus
Modulators of TCR signaling and T cell activation
regulation of binding
positive regulation of catabolic process
regulation of cellular response to stress
regulation of RNA splicing
regulation of cellular macromolecule biosynthetic process
Neutrophil degranulation
chromatin organization
HBV+#1_bef HBV+#2_bef HBV-#1_bef HBV+#2_aft HBV+#1_aft HBV-#1_aft
CD4+ TEFF
cell activation
Cytokine Signaling in Immune system
VEGFA-VEGFR2 signaling pathway
Signaling by Rho GTPases
Adaptive Immune System
Salmonella infection
Cellular responses to stress
regulation of T cell activation
Diseases of signal transduction by growth factor receptors and second messengers
Amyotrophic lateral sclerosis
Modulators of TCR signaling and T cell activation
regulation of apoptotic signaling pathway
Epstein-Barr virus infection
cellular response to cytokine stimulus
positive regulation of catabolic process
regulation of RNA splicing
regulation of cellular response to stress
regulation of cellular macromolecule biosynthetic process
Neutrophil degranulation
chromatin organization
HBV+#1_bef HBV+#2_bef HBV-#1_aft HBV-#1_bef HBV+#1_aft HBV+#2_aft
CD8+ TN
cell activation
Cytokine Signaling in Immune system
VEGFA-VEGFR2 signaling pathway
Signaling by Rho GTPases
Adaptive Immune System
Salmonella infection
regulation of cellular response to stress
Cellular responses to stress
positive regulation of catabolic process
Modulators of TCR signaling and T cell activation
Epstein-Barr virus infection
Diseases of signal transduction by growth factor receptors and second messengers
regulation of RNA splicing
regulation of apoptotic signaling pathway
cellular response to cytokine stimulus
Amyotrophic lateral sclerosis
regulation of T cell activation
regulation of cellular macromolecule biosynthetic process
Neutrophil degranulation
chromatin organization
HBV+#1_bef HBV-#1_bef HBV+#1_aft HBV+#2_aft HBV+#2_bef HBV-#1_aft
CD8+ TM
Diseases of signal transduction by growth factor receptors and second messengers
Epstein-Barr virus infection
Amyotrophic lateral sclerosis
regulation of T cell activation
cellular response to cytokine stimulus
positive regulation of catabolic process
regulation of RNA splicing
regulation of apoptotic signaling pathway
Membrane Trafficking
Neutrophil degranulation
Adaptive Immune System
Cytokine Signaling in Immune system
Salmonella infection
Signaling by Rho GTPases
VEGFA-VEGFR2 signaling pathway
cell activation
Cellular responses to stress
regulation of cellular macromolecule biosynthetic process
regulation of cellular response to stress
chromatin organization
HBV+#1_bef HBV-#1_bef HBV+#2_bef HBV-#1_aft HBV+#1_aft HBV+#2_aft
CD8+ TEFF

Fig S3. Feature change of T subsets pre- and post-anti-PD-1 therapy.

A. Bar plots of the number of DEGs (Bonferroni-adjusted P-value < 0.05 and average Log_2(fold change) (avg_log2FC) > 0.2) between pre- and post-anti-PD-1 therapy in each patient for each of the 6 T cell subsets.

B. Heatmap of enriched GO terms in each of the 6 T cell subsets.

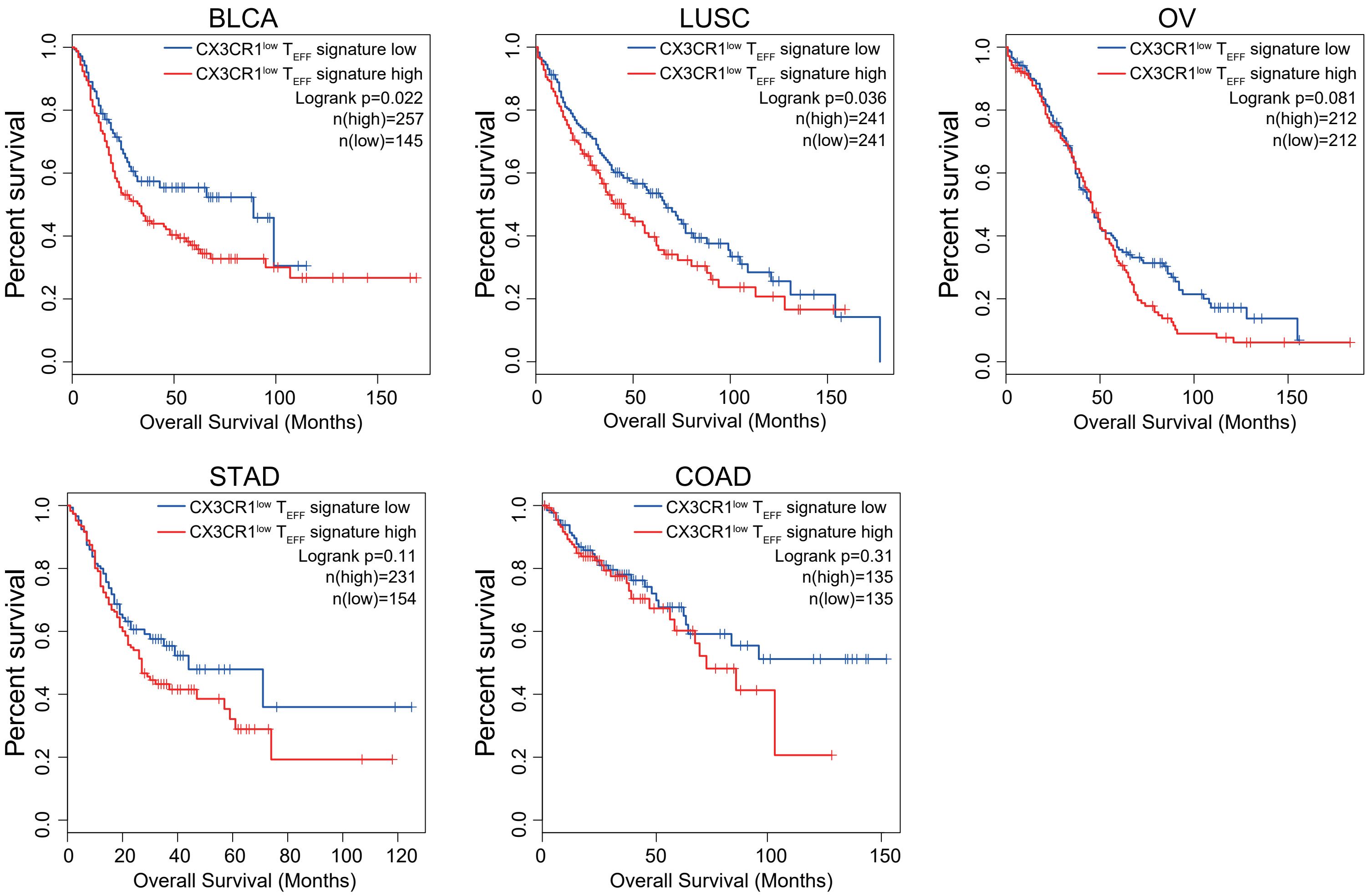

Fig S4. Survival curves of CX3CR1^{low} T$_{EFF}$ signature high patients and other patients in different tumor types from TCGA. P-value was determined by two-tailed log-rank test.

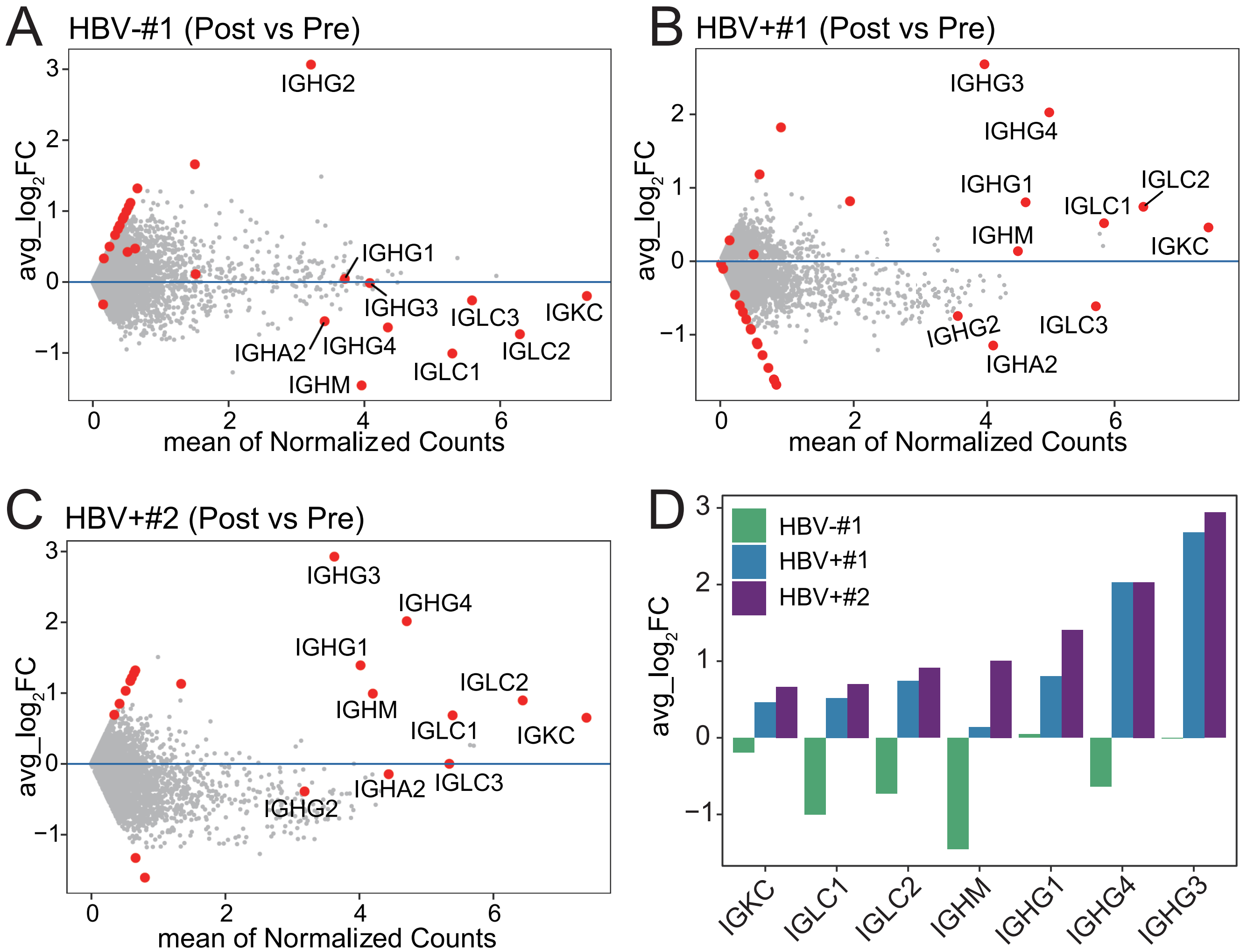

Fig S5. Change of B cell features pre- and post-anti-PD-1 therapy.

A-C. MA plot of gene expression of plasma cells in the three patients, HBV-#1 (A), HBV+#1 (B), HBV+#2 (C). Immunoglobulin gene was represented by red dot.

D. Bar plots of expression fold change of each of the 7 immunoglobulin genes in each patient.

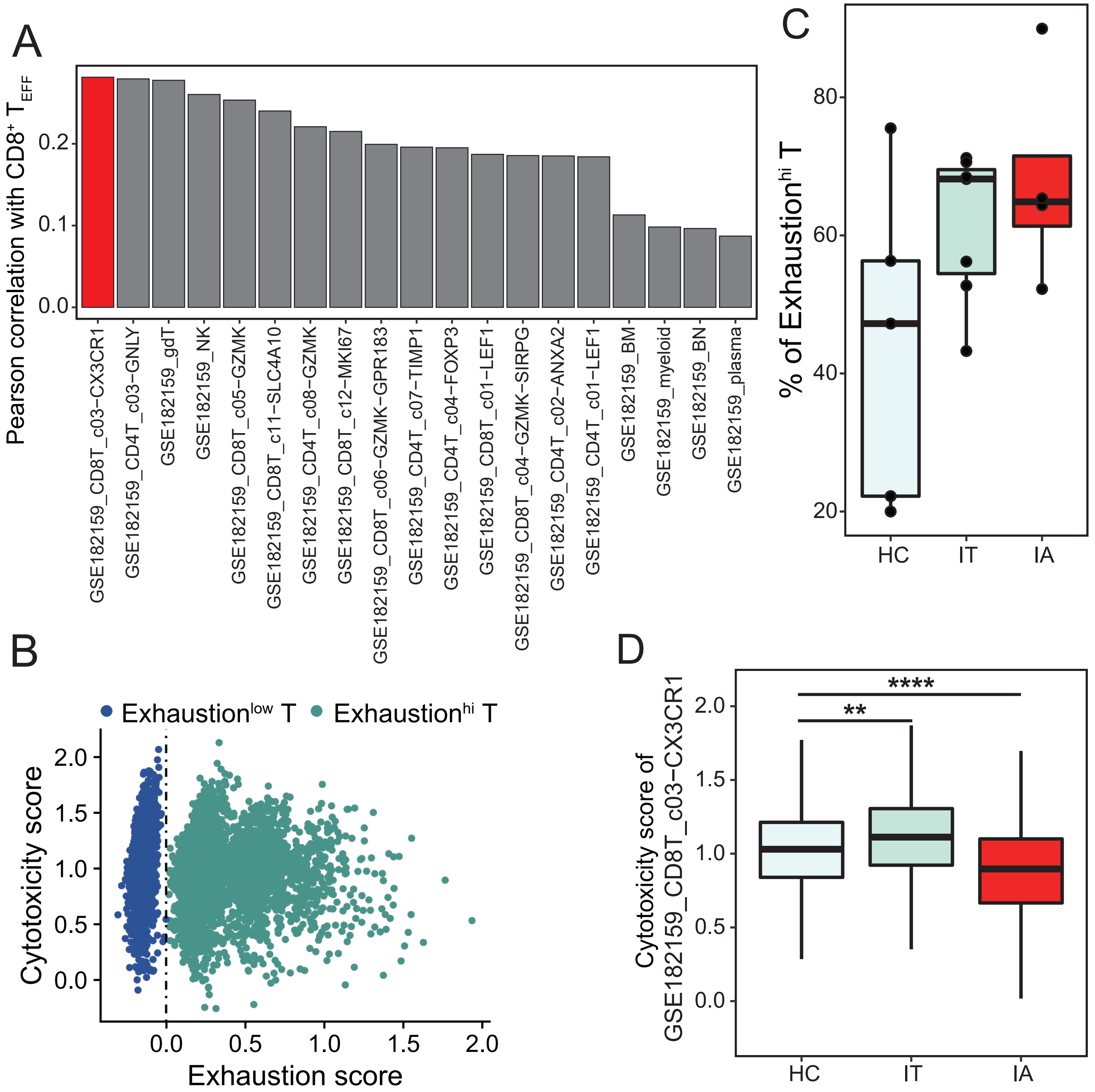

Fig S6. HBV positive patients had stronger immunosuppression than healthy individuals.
A. Pearson correlation between cell subsets in GSE182159 and CD8$^+$ T$_{EFF}$ of our study.
B. Scatter plot of the cytotoxicity score and exhaustion score of GSE182159_CD8T_c03−CX3CR1 showed two distinct subsets, namely Exhaustion^{hi} T and Exhaustion^{low} T. Vertical dashed line represents exhaustion score=0. HC, healthy control. IT, immune tolerant. IA, Immune Activation.
C. Box plot of the fraction of Exhaustion^{hi} T in GSE182159_CD8T_c03−CX3CR1.
D. Box plots of the cytotoxicity score of GSE182159_CD8T_c03−CX3CR1. P-value was determined by Student's t-test. HC, healthy control. IT, immune tolerant. IA, Immune Activation.
